# Effect of Continuous Lipopolysaccharide Induction on Oxidative Stress and Heart Injury in Weaned Piglets

**DOI:** 10.3390/vetsci12040330

**Published:** 2025-04-03

**Authors:** Jinyan Li, Guotong Zhao, Jin Liu, Xiaofen Hu, Wanting Yu, Jue Wang, Shengwei Zhong, Wenlu Zhu, Tingyu Yang, Yunxiao Zhou, Yijie Jiang, Lingna Bai, Mengyan Tu, Quan Yang, Yong Li

**Affiliations:** 1 College of Animal Science and Technology, Jiangxi Agricultural University, Nanchang 330045, China; 17590939039@163.com (J.L.); zhaogt_9439@163.com (G.Z.);; 2 Dezhou Agricultural and Rural Bureau, Dezhou 253000, China; 3 College of Veterinary Medicine, Shanxi Agricultural University, Jinzhong 030801, China

**Keywords:** cardiac injury, lipopolysaccharide, oxidative stress, weaned piglet

## Abstract

After weaning, piglets no longer consume breast milk, and their immune system is not yet fully developed. At this time, if weaned piglets are infected with *E. coli*, their subsequent growth will be seriously affected. In the present study, 48 healthy 28-day-old weaned piglets (6.65 ± 1.19 kg, Duroc × Landrace × Large White) were randomly divided into an LPS group and control group. Piglets in the LPS group were intraperitoneally injected with an LPS solution (LPS was dissolved in sterile saline to form a solution of 100 μg/mL and injected at a dose of 1 mL per kilogram of body weight) for 13 consecutive days. Piglets in the control group were injected with the same volume of sterile saline. On days 1, 5, 9, and 13 of the experiment, six piglets from each group were randomly selected for dissection, the blood and heart samples were collected, and then cardiac function-related indicators were detected. A portion of the heart tissue was fixed in 4% paraformaldehyde and further used to make paraffin sections; then, hematoxylin–eosin (H&E) staining was performed. Masson staining was used to detect the changes in collagen fibers in the hearts. The other parts of the heart tissues were frozen in liquid nitrogen and stored in a refrigerator at −80 °C for the detection of tissue antioxidant indices. The mRNA expression levels of the toll-like receptor 4 (TLR4) signaling pathway, transforming growth factor-β (TGF-β) signaling pathway, and inflammatory cytokines in heart tissues were detected by real-time PCR. The results showed that catalase (CAT) and superoxide dismutase (SOD) contents in the heart tissue homogenates increased significantly on days 1 and 5 in LPS-induced piglets (*p* < 0.01, *p* < 0.05), while total antioxidant capacity (T-AOC) and glutathione peroxidase (GSH-Px) contents decreased significantly on day 5 (*p* < 0.05). On day 5, the contents of serum cardiac function indicators lactate dehydrogenase (LDH), creatine kinase isoenzymes (CK-MB), and cardiac troponin I (cTn-I) were significantly increased in LPS-induced piglets (*p* < 0.01). On the 1st and 5th days, the heart tissue showed obvious pathological damage, which was manifested as the disordered arrangement of myocardial fibers, depression of myocardial cells, infiltration of inflammatory factors, congestion of capillaries, and significant increase in cardiac collagen fibers. On the 1st day, the mRNA expression levels of tumor necrosis factor-alpha (TNF-α) and interleukin 6 (IL-6) were significantly increased in LPS-induced piglets with heart injury (*p* < 0.01). On the 5th day, the mRNA expression levels of the TLR4 signaling pathway [TLR4, myeloid differentiation primary response gene 88 (MyD88), nuclear factor kappa-B (NF-κB)], TNF-α, and interleukin 10 (IL-10) were also significantly increased in LPS-induced piglets with heart injury (*p* < 0.01, *p* < 0.05). The mRNA expression levels of the TGF-β signaling pathway (TGF-β, Smad2, and Smad4) in cardiac fibrosis-related genes were significantly increased on days 5 and 9 (*p* < 0.01, *p* < 0.05). The mRNA expression levels of Smad3 and Smad7 in cardiac fibrosis-related genes were also significantly increased on day 9 (*p* < 0.01). These results indicate that oxidative stress occurs in the heart tissue of LPS-induced piglets on the 1st and 5th days, leading to cardiac tissue damage. However, on the 9th and 13th days, the degree of heart damage in the piglets was less than that on the 1st and 5th days, which may be due to the tolerance of piglets’ tissues and organs because of multiple same-dose LPS stimulations.

## Simple Summary

*E. coli* infection is common in pig farms, but weaning or weaned piglets are most affected. In this study, a stress model of weaned piglets was established by a continuous low-dose intraperitoneal injection of lipopolysaccharide (LPS), and the effect on the hearts of weaned piglets was explored. Serum cardiac function indices were detected in weaned piglets, and the antioxidant capacity, tissue structure, and tissue damage-related gene mRNA expression of the heart were analyzed. It was found that the heart damage of piglets induced by LPS was mainly concentrated in the early stage. Therefore, it is suggested that measures should be taken to prevent and treat Gram-negative bacterial infection in piglets at the early stage of breeding. These results also provide new evidence for comprehensively understanding the changes in tissue and organ damage caused by Gram-negative bacterial infections in weaned piglets.

## 1. Introduction

Pigs are artiodactyl mammals that are phylogenetically similar to primates, can survive in most types of environments, and are anatomically, physiologically, and genetically similar to humans. As a large animal model, pig models play an important role in understanding the progression of human diseases (especially metabolic and infectious diseases), discovering and validating therapeutic drugs, and evaluating the efficacy and toxicity of drugs [1]. Tang et al. [2] showed that weaning is one of the most critical periods in pig production, which is related to the overall economic benefit of the pig farm. Piglets in large-scale farms often suffer from weaning stress due to the nutrient and energy supply gap caused by abrupt weaning, the undeveloped immune system, the disturbance of intestinal absorption capacity caused by separation from sows, changes in diet, living environment, uncontrolled inflammation, oxidative stress, and the stimulation of other stressors [3].

The primary function of the heart is to maintain the body’s blood circulation. The heart pumps blood to the whole body through the contraction and expansion of the heartbeat, providing oxygen and nutrients for the whole body. The heart’s blood-delivery function depends on cardiomyocytes, the extracellular matrix (for biochemical and structural support), fibroblasts (for production of extracellular matrix), endothelial cells, and electrical signaling systems, among others. Cardiomyocytes, fibroblasts, and endothelial cells are a source of cytokines, chemokines, and growth factors. These cells express receptors that recognize leukocyte products, including mast cell-derived TNF-α, which activates endothelial cells [4]; myocardial cell-derived IL-6 can activate neutrophils by expressing intercellular adhesion molecule-1 (ICAM-1) [5]. T cell-derived interleukin-17 stimulates cardiac fibroblasts [6]; fibroblast-derived colony-stimulating factor (CSF2) can induce the generation and aggregation of myeloid cells [7]. Macrophage-derived transforming growth factor-β (TGF-β), vascular endothelial growth factor (VEGF), and IL-10 promote collagen production, neovascularization, and the resolution of inflammation [8]. It has been reported that after cardiac stress, macrophages in the heart expand locally and recruit monocytes in the blood to transform into cardiac macrophages, which participate in inflammatory responses and the repair of heart injury [9]. It has been reported that the heart of healthy adult mice contains major white blood cells, including macrophages, neutrophils, B cells, and T cells [10]. The pericardium, which wraps the heart, contains a serosal fluid with leukocytes such as macrophages and B cells, as well as lymphocytes and mast cells in the pericardial adipose tissue [11].

After cardiac injury, TGF-β signaling is activated and mediates cardiomyocyte growth, the activation of myocardial fibrosis, inflammation, and extracellular matrix deposition [12]. An increased release of activated fibroblasts or decreased collagen degradation leads to the accumulation of fibroblasts caused by the expansion of cardiac extracellular matrix proteins. Cardiac injury signals stimulate the increased release of hormones and growth factors on the surface of cardiac fibroblasts. At the same time, fibrosis reduces the flexibility of cardiac tissue and causes myocardial physiological dysfunction, thereby exacerbating harmful effects on the heart. In addition, the increased collagen content disrupts the electrical connections between cardiomyocytes, leading to the occurrence of arrhythmias [13].

Lipopolysaccharide (LPS) is an important component of the outer membrane of Gram-negative bacteria. When Gram-negative bacteria infect the host, LPS is released into the host body, triggering a strong immune response and inflammatory response, and is a key factor in the pathogenesis of bacterial infection [14]. Studies have shown that LPS can activate the cardiac TLR4 signaling pathway after its release into the bloodstream. When it is out of control, it will cause multiple organ dysfunction, such as myocardial depression. LPS can inhibit cardiac function contraction, reduce ventricular pressure, reduce cardiac output, and redistribute the systemic circulation [15], leading to decreased intestinal blood perfusion and intestinal mucosal ischemia. Intestinal hypoperfusion further disrupts the intestinal mucosa, which leads to increased intestinal permeability, bacterial translocation, and circulating lipopolysaccharide [16]. It has been reported that LPS can also mediate myocardial ischemia–reperfusion injury through the NLRP3 inflammasome [17]. Cardiac ischemia–reperfusion results in a marked decline in cardiac function, a decrease in left ventricular pressure, and a decrease in cardiac TNF-α levels during ischemia but an increase after perfusion [18]. High levels of TNF-α expression can lead to reduced myocardial systolic function and induce cell apoptosis [19]. Oxidative stress triggered by cardiac injury can trigger PKC-δ in mitochondria, and the activation of PKC-δ inhibits ATP regeneration, while cardiac mitochondrial inhibition leads to the generation of more reactive oxygen species (ROS) and the accumulation of reactive aldehydes (such as 4-hydroxynonenal, methylglyoxal, etc.), which will produce toxicity to a certain extent [20]. However, the specific effects of LPS on heart injury and oxidative stress in weaned piglets have not been fully studied. The aim of this study was to investigate the effects of continuous LPS induction on oxidative stress and cardiac injury in weaned piglets and to reveal the underlying mechanisms. Therefore, we used repeated intraperitoneal injections of LPS to stimulate weaned piglets to induce a stress model, to simulate the influence of piglets’ heart tissue under the infection of Gram-negative bacteria in breeding production, and to explore the damage of the heart tissue of weaned piglets. This study provides a scientific theoretical basis for clarifying the pathogenicity of Gram-negative bacterial infections in weaned piglets.

## 2. Materials and Methods

### 2.1. Experimental Animals

A total of forty-eight 28-day-old healthy weaned piglets (6.65 ± 1.19 kg, Duroc × Landrace × Large White) were purchased from Jiangxi Aoyun Agricultural Development Co., Ltd. (Nanchang, Jiangxi, China), and were pre-fed in the experimental animal house for three days, with free access to feed consisting of a basal diet and water. Then, these piglets were numbered consecutively and randomly divided into two groups: the LPS group and the control group. The weight of the piglets was recorded each morning before feeding; then, the LPS group was injected intraperitoneally with LPS (*Escherichia coli* serotype 055: B5; Cat. No. L2880; Sigma Chemical Inc., St. Louis, MO, USA) solution (LPS was dissolved in sterile saline to form a solution of 100 μg/mL and injected at a dose of 1 mL per kilogram of body weight per day), while the control group was injected with an equal amount of sterile saline. The experiment lasted for 13 days; on the 1st (D1), 5th (D5), 9th (D9), and 13th (D13) days, following the injection of LPS for 6 h, six piglets were randomly selected from the LPS and control groups, respectively, and anterior vena cava blood was collected. Subsequently, the blood was centrifuged for 10 min (4 °C, 3000 rpm) to obtain serum, which was then subpackaged and stored in a refrigerator at −80 °C. Then, the piglets were anesthetized and bled to death, and their hearts were collected. A portion of the heart tissue was fixed in 4% paraformaldehyde, while another portion was rapidly frozen in liquid nitrogen and placed in a refrigerator at −80 °C for further analysis. All procedures in this experiment were approved by the Animal Care and Use Committee of Jiangxi Agricultural University (approval number JXAULL-2022006).

### 2.2. Serum Cardiac Injury Indicator Assay

The serum to be tested was transferred to a refrigerator at 4 °C in advance. The piglet serum, porcine creatine kinase isoenzyme (CK-MB, Cat. No. YJ354125, Shanghai Yuanju Biotechnology Center, Shanghai, China), porcine cardiac troponin I (cTn-I, Cat. YJ002417, Shanghai Yuanju Biotechnology Center, Shanghai, China), and lactate dehydrogenase (LDH, Cat. No. A020-2, Nanjing Jiancheng Bioengineering Institute Inc., Nanjing, Jiangsu, China) were placed at room temperature for 20 min before the assay. Subsequently, the assay was performed according to the assay kit. The optical density (OD) values of CK-MB, cTn-I, and LDH were then determined at 450 nm using a microplate reader (Varioskan Flash 3001, Thermo Fisher, Waltham, MA, USA). The obtained data were plotted on a standard curve, and the content of the above indicators in the serum was calculated.

### 2.3. Assessment of Antioxidant Level

The cardiac tissues (200 mg) from both groups were weighed accurately in a sterile centrifuge tube. The pre-cooled saline (1.8 mL) was then added at the same time, and the contents of the tube were placed in a tissue grinder (KZ-III-F, Wuhan Sevier Biotechnology Co., Ltd., Wuhan, Hubei, China), then centrifugated for 10 min (4 °C, 2500 rpm), after which the supernatant was carefully pipetted to obtain a 10% heart tissue homogenate. The protein concentration of the cardiac homogenate was determined using a BCA protein assay kit (Cat. No. G2026, Wuhan Sevier Biotechnology Co., Ltd., Wuhan, Hubei, China). The antioxidant indices including catalase (CAT, Cat. No. A007-1-1), total antioxidant capacity (T-AOC, Cat. No. A015-3-1), glutathione peroxidase (GSH-Px Cat. No. A005-1), superoxide dismutase (SOD, Cat. No. A001-3), and malondialdehyde (MDA, Cat. No. A003-1) were measured subsequently. These antioxidant enzyme kits were purchased from Nanjing Jiancheng Bioengineering Institute (Nanjing, Jiangsu, China).

### 2.4. Histomorphological Observations of Heart

Cardiac tissues were fixed in 4% paraformaldehyde for a minimum of 48 h. Tissue blocks were trimmed to the appropriate size and then rinsed in tap water for 8–12 h. Tissues processed in the previous step were thoroughly dehydrated in ethanol (70–100%) at increasing concentrations, after which tissues were transparent in xylene and embedded in paraffin [21]. The tissues were sectioned at 4–6 µm using a Leica RM2245 (LEICA Camera AG, Wetzlar, Germany), and treated with xylene and gradient concentrations of ethanol. The microstructure of heart was assessed using hematoxylin–eosin (H&E) staining, and the distribution of collagen fibers in cardiac tissues was assessed using Masson staining. The changes were analyzed under a light microscope and photographed using CellSens Dimension software (Olympus Ver.1.12, Tokyo, Japan).

### 2.5. RNA Extraction and Real-Time Quantitative PCR

Total RNA was extracted from cardiac tissues by the Trizol method using MagZol reagent (Cat. No. R4801-02, Guangzhou Magen Biotechnology Co., Ltd., Guangzhou, Guangdong, China), and then the purity and concentration of RNA were determined by a UV spectrophotometer (NONODROP 2000, Thermo Fisher Tech, Inc., Waltham, MA, USA). The tested RNA was used to synthesize cDNA using the FAST RT Reagent Kit with gDNA Eraser (Cat. No. RR092A, TaKaRa Biotechnology Co., Ltd., Tokyo, Japan). cDNA was synthesized in a two-step process: First, 2 μL of 8 × gDNA premix was mixed with 1 μg of RNA template (the RNA mass of all samples was 1 μg before the experiment), and then Rnase-free H_2_O was added to make up 16 μL, which was gently mixed and centrifuged to remove the genomic DNA after incubation for 2 min at 42 °C. Second, 4 μL of 5 × RT Premix was added to the 16 μL of the reaction solution from the previous step, and finally, the cDNA was synthesized by incubation at 37 °C for 10 min and inactivation at 85 °C for 5 s.

The quantification of relevant mRNA expression in the cardiac tissue was performed on an Applied Biosystems 7500 using TB Green Premix Ex Taq II (Cat. No. RR820A, TaKaRa Biotechnology Co., Ltd., Tokyo, Japan). The gene primer sequences are listed in Table 1. The fluorescence quantification reaction system was 20 μL, which contained 10 μL TB Green Premix Ex Taq II (2×), 0.8 μL PCR forward primer (10 μM), 0.8 μL PCR reverse primer (10 μM), 0.4 μL ROX Reference Dye II (50×), 2 μL cDNA template, and 6 μL RNase Free H_2_O, mixed gently and centrifuged under the following reaction conditions: pre-denaturation by running at 95 °C for 30 s, PCR reaction by running at 95 °C for 5 s and 60 °C for 34 s, and PCR reaction for 40 cycles. Three technical replicates were made for each reaction and the relative fold difference in gene mRNA expression was calculated using GAPDH as an internal reference and the 2^−∆∆CT^ method [22].

### 2.6. Statistical Analysis

Relevant data were collated using Excel (version 2021, Microsoft, Redmond, WA, USA) and then analyzed to compare the differences between two groups by a one-way ANOVA through SPSS statistical software (version 25, IBM, Armonk, NY, USA), with probability values of ≤ 0.05 indicating a significant difference [23]. The final collated data were expressed as mean ± standard deviation and corresponding graphs were produced using Graph Prism 8.0 software (GraphPad, San Diego, CA, USA).

## 3. Results

### 3.1. Changes in Serum Biochemical Indices

**Figure 1:**
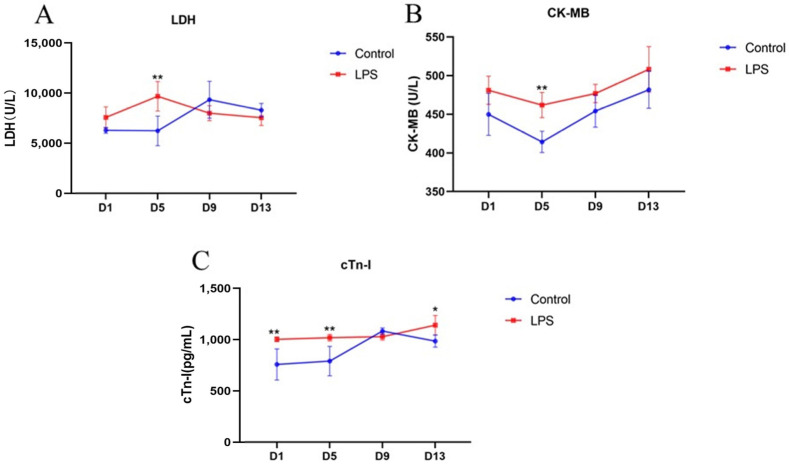
Dynamics of serum cardiac injury indices in weaned piglets between two treatments. Note: (**A**) LDH, lactate dehydrogenase; (**B**) CK-MB, creatine kinase isoenzyme MB; (**C**) cTn-I, cardiac troponin I. “*” indicates a significant difference (*p* < 0.05), “**” indicates an extremely significant difference (*p* < 0.01). Data are mean ± standard deviation (*n* = 6 in each group).

Serum levels of LDH, CK-MB, and cTn-I may reflect cardiac injury. The changes in serum levels of LDH, CK-MB, and cTn-I in weaned piglets at different days of LPS induction are shown in Figure 1. On the 5th day of LPS induction, the levels of LDH (Figure 1A, *p* < 0.01), CK-MB (Figure 1B, *p* < 0.01), and cTn-I (Figure 1C, *p* < 0.01) were significantly increased compared with the control group. At the same time, the levels of cTn-I (Figure 1C, *p* < 0.01, *p* < 0.05) were also significantly higher in the LPS group on the 1st and 13th day compared to the control group. However, the levels of LDH and CK-MB on the 1st, 9th, and 13th days of LPS induction and the level of cTn-I on the 9th day were not significantly different from those in the control group.

### 3.2. Changes in Cardiac Antioxidant Levels

The results of antioxidant levels in piglets’ heart tissues are shown in Table 2. The levels of CAT (*p* < 0.01, *p* < 0.05) and SOD (*p* < 0.01, *p* < 0.05) in the heart tissues of the LPS group were significantly higher on the 1st and 5th days, but the levels of T-AOC (*p* < 0.01, *p* < 0.05) were significantly lower in the heart tissues of the LPS group compared with the control group on the 1st and 5th days. Also, on the 5th day, the level of GSH-Px (*p* < 0.05) was significantly decreased in the heart tissue of the LPS group. Interestingly, throughout the experiment, no significant difference was found between the levels of MDA in the heart tissue of both groups.

### 3.3. Histomorphological Observations of Heart

#### 3.3.1. Histology Assessment

**Figure 2:**
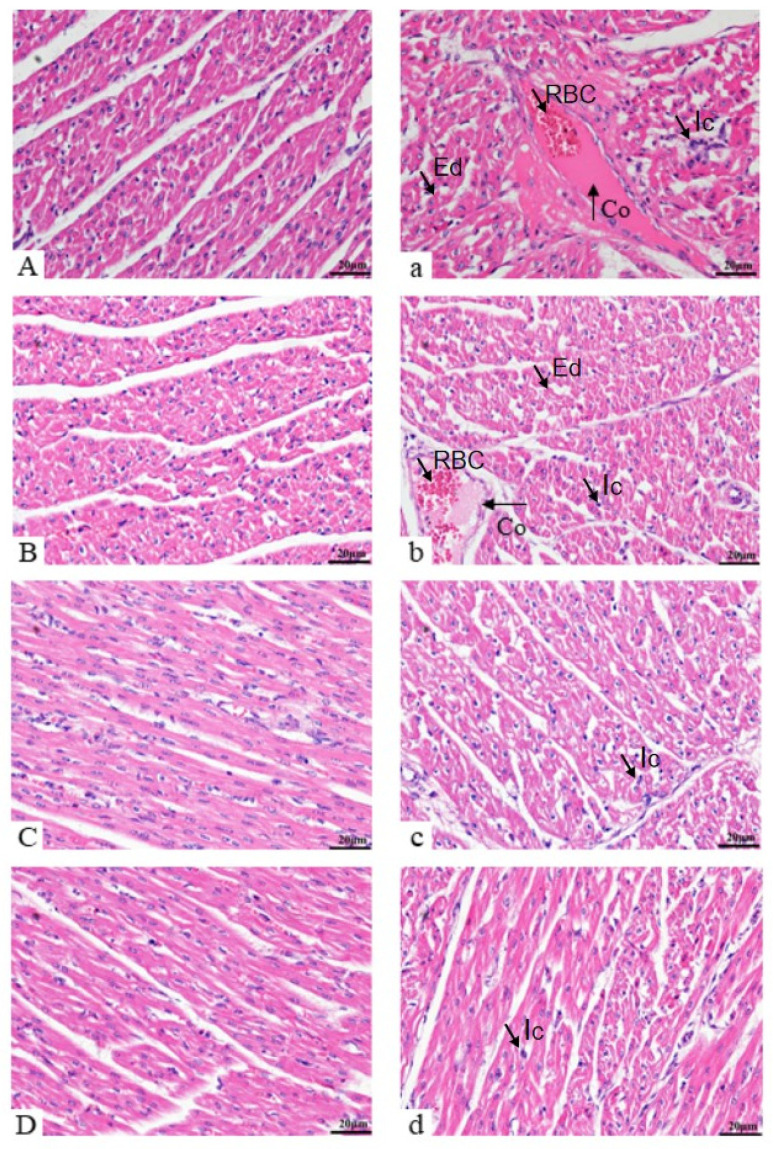
The microstructure changes of the heart in weaned piglets between two treatments. Note: Magnification = 400×, scale bar = 20 µm. Co indicates congestion; Ic indicates inflammatory cell infiltration; Ed indicates edematous; RBC indicates red blood cell. (**A**–**D**) indicates saline group, injected with sterile saline on D1, 5, 9, and 13, respectively; (**a**–**d**) indicates LPS group, injected with LPS on D1, 5, 9, and 13, respectively.

According to the results (Figure 2), the fibers of the myocardial tissue in the saline-injected control group were neatly arranged and the transverse stripe was clearer (Figure 2A–D). On the 1st and 5th days, the myocardial fibers of the LPS group were disorganized, myocytes were depressed or absent, inflammatory cell were infiltrated, capillaries were dilated and congested, and myocardial tissues were edematous (Figure 2a,b); in contrast, on the 9th and 13th days, the disorganization of the myocardial fibers of the LPS group tended to converge to that of the control group (Figure 2c,d).

#### 3.3.2. Cardiac Fiber Evaluation

**Figure 3:**
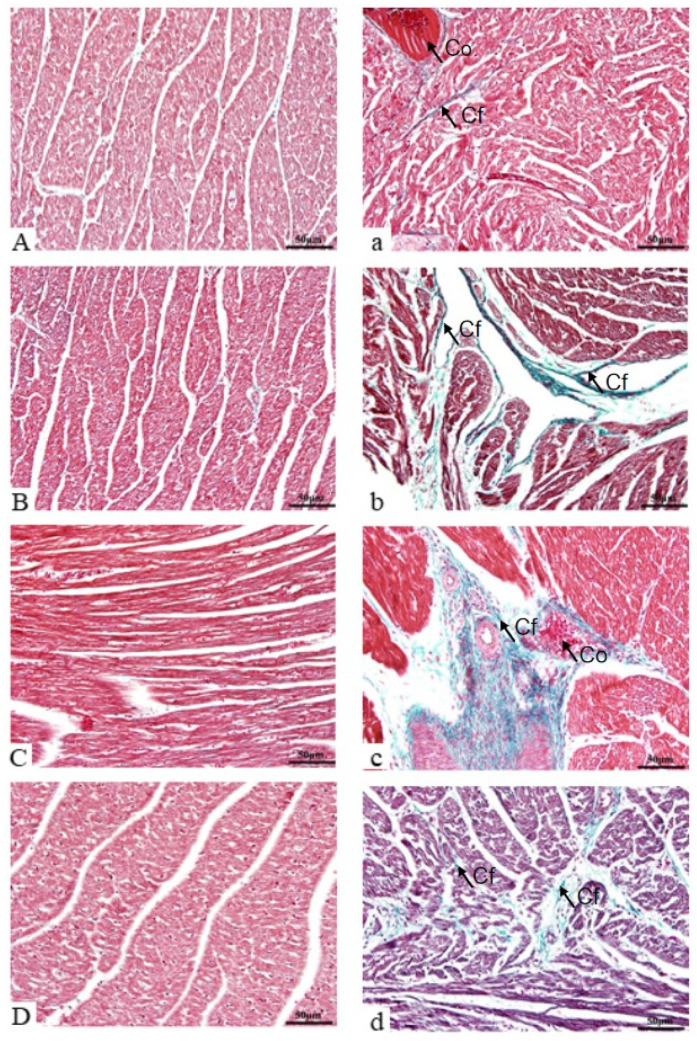
The collagen fiber changes in the hearts of weaned piglets between two treatments. Note: Magnification = 200×, scale bar = 50 µm. Co indicates congestion; Cf indicates collagen fibers. (**A**–**D**) indicates the saline group, injected with sterile saline on D1, 5, 9, and 13, respectively; (**a**–**d**) indicates the LPS group, injected with LPS on D1, 5, 9, and 13, respectively.

According to the results of cardiac fibrosis (Figure 3), the morphology and structure of cardiomyocytes were intact in the hearts from the control group, the nuclei were located in the center of the cells, and the fibers of the cardiomyocytes were neatly arranged (Figure 3A–D). On the 1st day of LPS induction, the myocardium of the LPS group was structurally incomplete, the capillaries were congested and dilated, and a small amount of green collagen fibers were deposited (Figure 3a); meanwhile, on the 5th and 9th days, the hearts of the LPS group also showed large green collagen fiber deposits accompanied by capillary congestion, and the area of cardiomyocytes was reduced and replaced by mesenchymal components (Figure 3b,c); and on the 13th day, the LPS group still showed large collagen fiber deposits accompanied by capillary congestion, and the area of cardiomyocytes was reduced and replaced by mesenchymal components (Figure 3b,c). On the 13th day of LPS induction, the LPS group still showed large green collagen fiber deposits among myocardial fibers, but the area was reduced compared with that on the 5th and 9th days (Figure 3d).

### 3.4. Gene mRNA Expression in the Heart

#### 3.4.1. TLR4 Pathway

**Figure 4:**
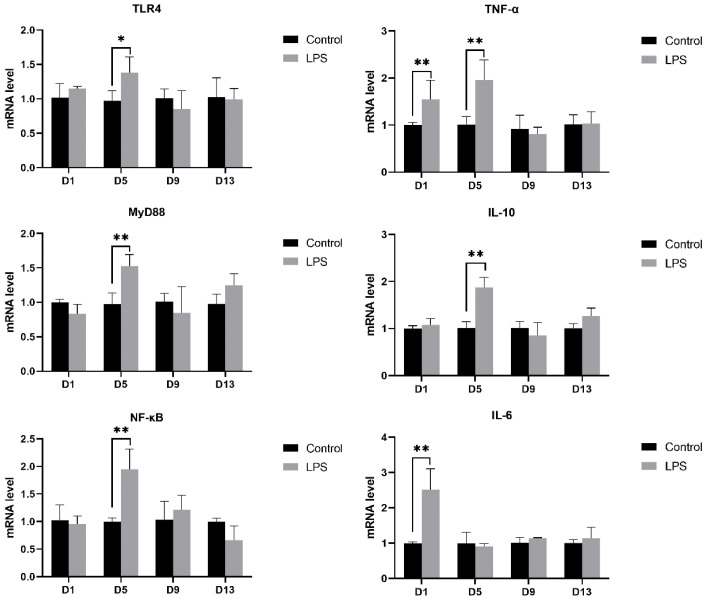
Effect of LPS induction on TLR4 pathway and inflammatory cytokine mRNA expression in the heart of weaned piglets between two treatments. Note: TLR4, toll-like receptor 4; MyD88, myeloid differentiation factor 88; NF-κB, nuclear factor-κB; TNF-α, tumor necrosis factor-alpha; IL-6, interleukin-6; IL-10, interleukin-10. “*” indicates a significant difference (*p* < 0.05) and “**” indicates an extremely significant difference (*p* < 0.01). Data are mean ± standard deviation (*n* = 6 in each group).

To investigate the effects of LPS induction on cardiac injury and inflammatory cytokines in weaned piglets, the mRNA expression of TLR4, MyD88, NF-κB, TNF-α, IL-10, and IL-6 genes was measured, and the results are shown in Figure 4. The mRNA expression of TLR4, MyD88, NF-κB, TNF-α, and IL-10 was significantly increased on the 5th day of LPS induction (TLR4, *p* < 0.05; MyD88, *p* < 0.01; NF-κB, *p* < 0.01; TNF-α, *p* < 0.01; IL-10, *p* < 0.01), while on the 1st day, the mRNA expression of TNF-α and IL-6 was also significantly increased (TNF-α, *p* < 0.01; IL-6, *p* < 0.01). However, there was no significant difference between the LPS and control groups on both the 9th and 13th days.

#### 3.4.2. TGF-β Pathway

**Figure 5:**
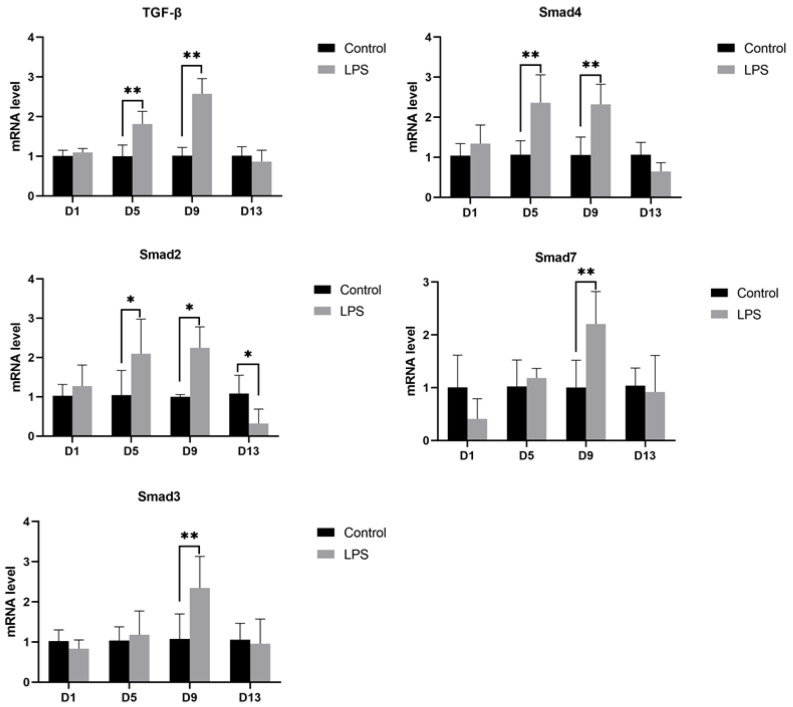
Effects of LPS induction on the TGF-β-related mRNA expression in the heart of weaned piglets between two treatments. Note: TGF-β, transforming growth factor β; Smad2, SMAD family member 2; Smad3, SMAD family member 3; Smad4, SMAD family member 4; Smad7, SMAD family member 7. “*” indicates a significant difference (*p* < 0.05) and “**” indicates an extremely significant difference (*p* < 0.01). Data are mean ± standard deviation (*n* = 6 in each group).

To investigate the effect of LPS induction on cardiac fibrosis in weaned piglets, the mRNA expression of TGF-β, Smad2, Smad3, Smad4, and Smad7 genes was determined, and the results are shown in Figure 5. On the 5th and 9th days of LPS induction, the mRNA expression of TGF-β, Smad2, and Smad4 was significantly increased (TGF-β, *p* < 0.01; Smad2, *p* < 0.05; Smad4, *p* < 0.01). On the 9th day of LPS induction, the mRNA expression of Smad3 and Smad7 was significantly increased (Smad3, *p* < 0.01; Smad7, *p* < 0.01). Interestingly, the mRNA expression of Smad2 was significantly reduced (Smad2, *p* < 0.05) on the 13th day of LPS induction. Except for Smad2, the TGF-β pathway genes were not significantly different between the LPS and control groups on the 1st and 13th days.

## 4. Discussion

The contraction of the heart drives blood that is rich in nutrients and oxygen to the body, continuously providing energy and maintaining homeostasis throughout the body. Once bacteria and bacterial metabolites enter the blood and flow through the heart through blood circulation, they may trigger an inflammatory response in the heart [24]. Missov et al. [25] first described cTn-I as a biochemical marker of cardiomyocyte injury. LDH and CK-MB are also commonly used as markers of cardiac injury [26]. Among them, LDH can convert lactate to pyruvate, so that the heart can use lactate as a metabolic substrate, which eliminates the possibility of myocardial fatigue due to the pH imbalance [27]. CK-MB is one of the isoenzymes of CK, which is associated with mitochondria and cytosol in muscle cells, and about 20% of CK in the myocardium is in the form of myoglobin, which provides sensitivity and specificity for the diagnosis of acute myocardial injury [28]. Troponin (cTn) is mainly composed of the calcium-binding subunit troponin C, the tropomyosin-binding subunit troponin T, and the inhibitory subunit troponin I (cTnI) [29]. The action potential triggered by the working heart causes an increase in Ca^2+^ in cardiomyocytes, which bind to cTn and induce a conformational change in cardiac thin myofilaments, which bind myosin and actin tightly and generate contractility. During cardiac relaxation, Ca^2+^ dissociates from cTn as the intracellular Ca^2+^ level decreases. The interaction between actin and myosin would in turn be blocked by cTn [30]. Therefore, the content of cTnI can be used as an early detection index of cardiac injury. Studies have shown that the levels of LDH, CK-MB, and cTn-I in serum increase when the heart is damaged [31], and these three indicators also decrease.

Several previous studies have shown that LPS stimulation can induce heart injury and significantly increase the expression levels of LDH, CK-MB, and cTn-I in serum. In this study, serum cTn-I levels in weaned piglets were significantly higher than those in the control group except the 9th day of LPS induction, indicating that its sensitivity to heart injury was different from LDH and CK-MB. This result is consistent with the above reported claim that “cTn-I can be used as an early detection index of heart injury”. It is indicated that there is value in detecting serum cTn-I in the early biochemical in-dicators of cardiac function damage. The serum cTn-I levels of weaned piglets were significantly increased on the 5th day, and the serum LDH and CK-MB levels were also significantly increased during the same period, which may indicate that the cardiac function damage reached its peak in this period among the four periods measured in the whole experiment. On the other hand, from the 9th to 13th days, the serum LDH content of piglets was not significantly different from that of the control group, and the serum CK-MB content also showed a similar change, but the serum CK-MB content of piglets in the LPS group still showed an upward trend, although it was not significant. However, similar to the significant increase in the serum cTn-I level in the LPS group on 13th day, this may be related to the irreversibility of myocardial injury (on the 9th and 13th days, there were no significant differences in the three indicators compared with the control group, but the cTn-I on day 13 excluded, indicating that the heart function gradually changed from an injury state to a repair state. However, some irreversibly regenerated cardiomyocytes have been lost, which will still affect the physiological function of the heart). After myocardial tissue injury (due to factors such as ischemia, trauma, toxic injury, or inflammation), cTn-I levels in the peripheral blood are elevated despite normal levels of CK-MB activity [32]. It has been reported that when the heart responds to infection, a large number of immune cells in the heart or the body’s circulation will be recruited to the heart to remove damaged cardiomyocytes and tissues, eliminate invasive sources, and promote healing. Although these benefits have been observed, in some cases, immune cells can cause irreversible tissue damage [33]. In conclusion, compared with the control group, the levels of LDH, CK-MB, and cTn-I in the serum of weaned piglets were significantly increased after multiple LPS inductions. These experimental results are consistent with the literature reports, indicating that LPS induction caused cardiac function damage in piglets.

The state of oxidative stress reflects the imbalance between oxidants (mainly oxygen free radicals) and antioxidants produced in the body. The excessive production of reactive oxygen free radicals (ROS) can cause the oxidative modification of cellular macromolecules (such as DNA, lipids, proteins, etc.) and lead to the accumulation of damaged macromolecules, inducing tissue damage. The simultaneous overproduction of ROS may directly inhibit the activity of antioxidant enzymes, such as superoxide dismutase (SOD) and catalase (CAT), or deplete antioxidant molecules, such as glutathione (GSH) [34]. Gram-negative bacteria invade the body and proliferate and produce a large amount of LPS, which induces a rapid defense response in the body and leads to a rapid increase in the concentration of ROS. At the same time, the intestinal barrier is damaged and the intestinal permeability is increased. Excessive LPS can also be transferred to the blood and affect other tissues and organs [35]. Bi et al. [36] used Escherichia coli LPS to successfully construct an oxidative stress model of broilers. In addition to oxidative stress, LPS can also induce inflammation, growth inhibition, a variety of diseases, and even death [37], all of which indicate that LPS can induce oxidative stress in tissues and organs of the body. Malondialdehyde (MDA), reactive oxygen species (ROS), and 8-hydroxy-2-deoxyguanosine (8-OHdG), as oxygen free radicals and their products, can reflect the level of oxidative damage in tissues [38]. Indicators such as total antioxidant capacity (T-AOC), SOD, CAT, glutathione peroxidase (GSH-Px), and GSH also show tissue antioxidant levels [39]. The body contains a large number of antioxidants, including antioxidant molecules and enzymes, which can remove various reactive oxygen species and prevent the generation of oxidative stress induced by reactive oxygen species [40]. T-AOC reflects the overall levels of enzymatic and non-enzymatic antioxidants in the body [41], in which SOD dismutates superoxide to hydrogen peroxide, which is further degraded by CAT and GSH-Px [42]. According to the results of this experiment, LPS induction can significantly reduce the activity of T-AOC in the heart, and the changes mainly occur on the 1st and 5th days of LPS induction, indicating that the antioxidant level in the heart of piglets induced by LPS decreases during these two periods, leading to oxidative stress damage in tissues and organs. On the 1st and 5th days, the decrease in cardiac antioxidant levels was similar to the decrease in serum biochemical markers of cardiac function, which confirmed that significant damage occurred in cardiac tissue during these two periods. The MDA level did not change significantly in these hearts, which may be due to that the tissue damage and oxidative stress induced by LPS mainly occurred in the early stage of the experiment (1st and 5th days), and the sensitivity of the body to LPS was weakened after repeated induction. Serum MDA levels did not show statistically significant differences during the LPS challenge [43]; however, another study showed a significant increase in plasma MDA concentration after the LPS challenge [44], indicating the complexity behind the changes in MDA expression levels.

The structural integrity of tissues and organs is essential for their complete function. When cardiomyocytes are normal, myocytes are arranged neatly, the cell membrane is intact, and the nucleus is clear. Zhang Xh et al. [45] showed that LPS stimulation could cause myocardial structural disorder, muscle fiber rupture, and inflammatory cell infiltration in mice, and spermidine could alleviate myocardial injury in mice. In this study, H&E staining showed that the myocardium of weaned piglets induced by LPS showed a disordered arrangement of myocardial fibers, depression or loss of myocardial cells, infiltration of inflammatory factors, telangiectasia, and congestion. Later, the injury was attenuated, the myocardial space was reduced, and the inflammatory cell infiltration was reduced. These results showed that at the early stage of LPS induction, macrophages in the heart recognized LPS that entered blood circulation and started the corresponding signaling cascade, which triggered the increase in inflammatory cytokine expression and led to myocardial damage. In the later stage, with the body’s detoxification and tolerance to LPS stimulation, and the heart repair through fibrosis and other ways, the myocardial injury is relieved. Wang Yun et al. [46] investigated the effects of different doses of bupleurum on LPS-induced myocardial infarction in mice and found that bupleurum could effectively attenuate fibrosis induced by LPS and reduce collagen fiber deposition. In this experiment, Masson staining showed that the area of myocardial collagen fibers in the LPS group was significantly higher than that in the control group, indicating that LPS induced myocardial fibrosis in piglets. At the same time, the results showed that the area of collagen fibers first increased and then decreased, indicating that the heart was damaged after LPS stimulation, but had not yet converted to a repair state. After the peak of collagen deposition on the 9th day of LPS stimulation, the heart significantly expressed the growth factor TGF-β to repair the damage in the heart, but this repair may be limited in degree and last for a long time. This was evidenced by the large area of collagen deposition in the heart tissue on the 13th day. However, fibrosis is a double-edged sword. If excessive fibrosis is not effectively degraded, it will affect cardiac function. Zhuang et al. [47] used LPS to induce sepsis in mice, and found that the myocardial fibrosis and cardiac dysfunction occurred in mice.

TLR4 is one of the most widely studied toll-like receptor (TLR) family. TLR4 can be activated by LPS and trigger a pro-inflammatory response, thereby promoting the clearance of invasive bacteria and inducing tissue repair [48]. In the context of LPS-induced TLR4 proinflammatory signaling, NF-κB is continuously activated to control the production of proinflammatory factors such as TNF-α and IL-6, while leading to the expression of interferon (IFN) and IFN-induced chemokines such as IL-10. Studies have shown that low levels of TNF-α may have a protective effect on the heart by enhancing myocardial tolerance to ischemia [49], and TNF-α may inhibit the expression of inflammatory cytokines such as IL-1, IL-2, and IL-6 in the heart [50]. Xiao et al. [51] found that LPS can cause cardiac dysfunction, activate the TLR4/NF-κB signaling pathway, and increase the expression of inflammatory cytokines (TNF-α, IL-1β, IL-6). In this study, LPS induction increased the mRNA expression of these genes. The mRNA expression of TLR4, MyD88, NF-κB, TNF-α, and IL-10 in heart tissue was significantly increased on the 5th day after LPS induction, which was consistent with the trend of pathological changes in heart tissue. The significant increase in the expression of IL-10 mRNA may be due to myocardial injury and tissue wound repair. However, there were no significant changes on the 9th and 13th days. This may be due to the increase in CAT in the myocardial tissue [52], the anti-inflammatory factor IL-10 (IL-10 as an immunomodulatory cytokine can act as both antigen and anti-inflammatory factor) [53], and the decreased sensitivity of cardiac macrophages to LPS to produce endotoxin tolerance [54].

The excessive deposition of cardiac extracellular matrix (ECM) proteins leads to cardiac interstitial expansion and myocardial fibrosis, which is often accompanied by cardiac injury [55]. Activated fibroblasts are the main source of ECM proteins. Cardiac fibroblasts are mainly located in the endocardium and adventitia and can quickly activate and repair with a variety of collagen (such as type I collagen) and growth factors in the heart during injury [56]. TGF-β is the most typical fibrotic growth factor that induces and activates myocardial fibrosis [57]. There is a potential role of TGF-β in the normal heart, but it does not bind to the receptor; it is activated when myocardial injury occurs and repairs myocardial tissue. Also, it is noteworthy that TGF-β can cause diseases and regulate inflammation to fibrosis; inhibiting TGF-β-related receptors at different times of inflammation has different results [58]. However, the specific disruption of TGF-β receptors can produce a protective effect on cardiomyocytes [59]. TGF-β is secreted from cells by binding to the latency-associated peptide (LAP) to form a complex and then activated by plasmin. The activated TGF-β is activated by taking off the LAP and binding to the corresponding TGF-β receptor to transact the signal into cells. Phosphorylated Smad2/Smad3 forms a complex with Smad4 and is transported to the nucleus to activate or repress target gene transcription [60]. Among them, Smad6 and Smad7 competitively bind to the TGF-β receptor to block Smad2/Smad3 signaling and inhibit TGF-β signaling, while Smad4 keeps the Smad2/Smad3 complex active and facilitates signaling to the nucleus [61]. In the present study, the mRNA expression levels of TGF-β, Smad2, and Smad4 were significantly increased on the 5th and 9th days compared with the control group. Smad4 can assist Smad2/Smad3 signaling. Similar to Masson staining results (increased collagen fiber area) in the heart on the 5th and 9th days, Smad2 was significantly decreased on the 13th day. The analysis of oxidative markers and pathological changes showed that on the 13th day, the inflammatory response in the heart tissue was reduced, and the expression of TGF-β in the heart was decreased. On the 9th day, the mRNA expression levels of Smad3 and Smad7 were significantly higher than those of the control group to avoid excessive cardiac fibrosis. Combined with Masson staining results, it may be that the up-regulation of Smad7 gene expression inhibits the continued transmission of the TGF-β signaling pathway and reduces the continuation of cardiac fibrosis. These were similar to the results of cardiac Masson staining, indicating that LPS induction may induce cardiac fibrosis in piglets by affecting TGF-β related genes. At the same time, it also indicates that the process of cardiac fibrosis is not continuous and may be affected by various factors. Xu Zhemin et al. [62] found that the mRNA and protein expression of TGF-β in rat cardiac fibroblasts was significantly increased, and the mRNA expression level of type I collagen was also significantly increased, which was consistent with the present results.

## 5. Conclusions

In the early stage of multiple LPS inductions, oxidative stress occurs in the heart of piglets, which activates the TLR4 signaling pathway and increases the expression of related inflammatory factors, eventually leading to heart injury. In the mid-stage of LPS induction, the cardiac TGF-β signaling pathway is activated to increase the expression of myocardial collagen fibers to repair the injured myocardium. The reduced damage to the tissues and organs of weaned piglets in the late stage may be due to the attenuated response to LPS repeated induction. In conclusion, the early stage of LPS induction has a significant negative effect on the circulation system of piglets, which affects the system’s normal growth and development. Therefore, once piglets are infected by Gram-negative bacteria releasing LPS, the treatment and other measures should be taken at an early stage to reduce the damage to piglets.

## Figures and Tables

**Table 1:** Sequence of heart-related genes in piglets.

Gene	Primer Sequence (5′ → 3′)	Product Size (bp)	GenBank No.
GAPDH	F: TGACATCAAGAAGGTGGTGAAG	159	NM_001206359.1
	R: TTGACGAAGTGGTCGTTGAG		
TLR4	F: CTCCGGGTCACTTCTGTTCA	143	NM_001293317.1
	R: TAATGTTAGGAACCACCTGCAC		
MyD88	F: TGCCTTCATCTGCTACTGC	81	NM_001099923.1
	R: AGCCGATAGTTGGTCTGTTC		
NF-κB	F: TGCCAGACACAGATGACCG	195	NM_001114281.1
	R: ATGGCGTAAAGGGATAGGGC		
IL-6	F: ACCTGCTTGATGAGAATCACC	97	NM_214399.1
	R: CCTCGACATTTCCCTTATTGCT		
TNF-α	F: CCAATGGCAGAGTGGGTATG	116	NM_214022.1
	R: TGAAGAGGACCTGGGAGTAG		
IL-10	F: GAAGACGTAATGCCGAAGGC	121	NM_214041.1
	R: AGGGCAGAAATTGATGACAGC		
TGFB1	F: AAAGCGGCAACCAAATCTATGA	206	NM_214015.2
	R: GCTGAGGTAGCGCCAGGAAT		
SMAD2	F: TGTGTTACCATACCAAGGTCCC	100	NM_001256148.1
	R: GATCAGGCCAGCGCCATAAT		
SMAD3	F: ACGACTACAGCCATTCCATCC	110	NM_214137.1
	R: CTCTCCATCTTCACTCAGGTAGC		
SMAD4	F: CAGGACAGCACAGAATGGATT	110	NM_214072.1
	R: GGTGAGGCAAATTAGGTGGGTATG		
SMAD7	F: TACTGGGAGGAGAAGACGAGAGTG	241	NM_001244175.1
	R: TGGCTGACTTGATGAAGATGGG		

F: forward primer; R: reverse primer.

**Table 2:** Dynamic antioxidant levels in heart tissue of weaned piglets between two treatments.

Items	Group	D1	D5	D9	D13
CAT	control	4.98 ± 0.63	5.40 ± 0.30	6.70 ± 1.37	5.06 ± 0.56
(U/mL)	LPS	9.04 ± 0.85 **	8.40 ± 1.46 *	6.56 ± 1.42	5.61 ± 0.86
MDA	control	0.779 ± 0.217	0.898 ± 0.268	0.791 ± 0.268	0.647 ± 0.117
(nmol/mL)	LPS	0.838 ± 0.261	1.061 ± 0.575	0.891 ± 0.124	0.501 ± 0.044
SOD	control	42.22 ± 3.63	45.18 ± 2.88	46.05 ± 6	46.77 ± 0.37
(U/mL)	LPS	51.28 ± 2.32 **	53.36 ± 2.42 *	43.01 ± 5.24	43.7 ± 0.98
T-AOC	control	0.355 ± 0.041	0.327 ± 0.04	0.272 ± 0.006	0.298 ± 0.027
mM	LPS	0.285 ± 0.027 ^##^	0.269 ± 0.023 ^#^	0.287 ± 0.13	0.290 ± 0.026
GSH-Px	control	64.91 ± 5.86	75.42 ± 8.14	60.51 ± 5.53	64.31 ± 3.61
(U/mL)	LPS	70.93 ± 17.38	56.47 ± 5.89 ^#^	56.30 ± 5.61	66.64 ± 11.65

Note: CAT, catalase; MDA, malondialdehyde; SOD, superoxide dismutase; T-AOC, total antioxidant capacity; GSH-Px, glutathione peroxidase. “*” or “#” indicates a significant increase or decrease (*p* < 0.05), “**” or “##” indicates an extremely significant increase or decrease (*p* < 0.01). Data are mean ± standard deviation (*n* = 6 in each group).

## Data Availability

All data appear in the manuscript. For further inquiries, please contact the first author or corresponding author.
